# Pharmacokinetic study of mangiferin in rat plasma and retina using high-performance liquid chromatography

**Published:** 2010-08-17

**Authors:** Yunlong Hou, Shengjun Fan, Hong Zhang, Yuanqin Gu, Xuhui Yu, Baoxin Li

**Affiliations:** 1 Eye hospital, The First Affiliated Hospital of Harbin Medical University, Harbin, P.R. China; 2 Department of Pharmacology, Harbin Medical University, Harbin, P.R. China

## Abstract

**Purpose:**

Although the naturally occurring antioxidant mangiferin has been widely used, it is not yet known whether it can cross the blood-retina barrier (BRB) and enter the eye. The purpose of this experiment was to investigate the ability of mangiferin to pass the blood-retina barrier.

**Methods:**

Sprague–Dawley rats were used for biologic fluid sampling after intravenous administration of mangiferin at doses of 10, 25, and 50 mg/kg. Blood and retina samples were collected at different time points post-dose. High-performance liquid chromatography (HPLC) separation was conducted on a COSMOSIL 5C_18_—MS—II column (4.6 mm×250 mm, 5 μm) with a flow rate of 1.0 ml/min using a mobile phase comprised of methanol −2% glacial acetic acid (40:60 v:v).

**Results:**

The HPLC method has proven suitable to determine the presence of mangiferin in the eye. The plasma concentration of mangiferin was dose dependent. Pharmacokinetic parameters of mangiferin in plasma after intravenous administration were fitted to the two-compartment model with the first-order elimination and first-order transfer between central and peripheral compartments. The concentration of mangiferin in the retina goes with that in the blood. Mangiferin concentrations in the retina reached 5.69±1.48 μg/ml 0.5 h after intravenous administration (50 mg/kg) and then dropped gradually to 0.30±0.02 μg/ml 5.0 h later. The eye–to-plasma concentration ratio was 2.80%.

**Conclusions:**

Mangiferin can pass the blood-retina barrier after a single intravenous administration and may be a potential natural antioxidant in treating eye diseases.

## Introduction

**Figure 1:**
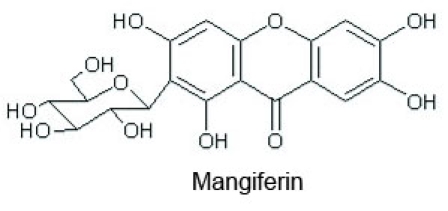
Chemical structure of mangiferin (1,3,6,7-tetrahydroxyxanthone-C-2-β-D-glucoside).

The protective role of naturally occurring antioxidants has recently been a focus of attention. Mangiferin, 1,3,6,7-tetrahydroxyxanthone-C-2-β-D-glucoside (Figure 1), is one such naturally occurring polyphenol [1] that is widely found in many herbs such as *Anemarrhena asphodeloides Bung* (Chinese herbal name: Zhi-Mu) [2] and *Mangifera indica* [3]. Due to its antioxidant [4], anti-apoptotic [5], immunomodulating [6] and anti-diabetic potential [7], mangiferin may have a putative role in treating certain eye diseases, such as age-related macular degeneration (AMD), cataracts and diabetic retinopathy [8,9]. However, whether mangiferin can pass the blood-ocular barrier remains unknown. Before mangiferin is applied in the treatment of eye diseases, it is necessary to know the pharmacokinetic parameters of mangiferin in the eye.

The eye is a unique organ with a blood-retina barrier (BRB) [10]. This barrier is relatively impermeable, like the blood–brain barrier (BBB), controls the passage of substances from the blood into the eye and normally keeps most drugs out of the eye [11]. Although many published papers have described the bioanalysis of mangiferin in plasma and urine [12–16], very few reports exist assessing the amount of mangiferin allowed into the eye. This paper investigated the ability of mangiferin to pass the BRB and provided a limited pharmacokinetic study of mangiferin in the eye.

## Methods

### Reagents and chemicals

Mangiferin and 4-Nitrophenol, which was used as an internal standard (I.S.), were purchased from Sigma (Sigma-Aldrich, Tokyo, Japan). The chromatographic solvents and reagents were obtained from the National Institute for the Control of Pharmaceutical and Biologic Products (Beijing, China). All substances were of chromatographic grade. De-ionized water was prepared using a Milli-Q water purifying system from Millipore Corp. (Bedford, MA).

### Animal treatment

Adult male Sprague–Dawley rats (280~320 g) were supplied by the Animal Research Center at Harbin Medical University (Harbin, Heilongjiang Province, China). The rats were specifically pathogen-free and housed in a limited access rodent facility for at least 5 days. The room controls were set to maintain the temperature at 22 °C±2 °C, the relative humidity at 55%±15%, the illumination intensity at 150~300 lx, an air ventilation frequency of 15~20 times/h, and a 12 h illumination period (07:00~19:00) each day. The animals drank sterilized drinking water, and standard chow diet was supplied ad libitum to each cage. The animal experiments were performed in accordance with the ARVO Statement for the Use of Animals in Ophthalmic and Vision Research, and were approved by the Animal Ethics Committee of Harbin Medical University.

### Preparation of standard and internal standard stock solutions

The standard stock solution was prepared by dissolving 10 mg of mangiferin in a 10 ml methanol to obtain a nominal concentration of 1,000 μg/ml. The internal standard (I.S.) stock solution was prepared by dissolving 0.20 mg of 4-Nitrophenol in 100 ml of methanol to obtain a nominal concentration of 2 μg/ml. All the stock solutions were maintained at 4 °C until use.

### Preparation of assay standard samples

Mangiferin standard samples of plasma (0.50, 1.00, 5.00, 10.00, 20.00, 50.00, 100.00, and 150.00 μg/ml) were prepared by spiking control rat plasma with appropriate amounts of the standard stock solution prepared above. Quality control (QC) samples were used to determine accuracy and precision of the method, and were independently prepared at low (1.00 μg/ml), medium (10.00 μg/ml) and high (100.00 μg/ml) concentrations in the same manner. The I.S. was added to each standard sample immediately before sample processing. All the samples were stored at −20 °C until analysis.

Mangiferin standard samples of eyes (0.10, 0.50, 1.00, 2.00, 4.00, 6.00, 8.00, and 10.00 μg/ml) were prepared by spiking control rat eye samples with appropriate amounts of the standard stock solution prepared above. QC samples were used to determine accuracy and precision of the method, and were independently prepared at low (0.50 μg/ml), medium (2.00 μg/ml) and high (8.00 μg/ml) concentrations in the same manner. The I.S. was added to each standard sample immediately before sample processing. All the samples were stored at −20 °C until analysis.

### Extraction procedure

Each rat (n=6) was fasted for 24 h with free water during the experiment. Then, they were intravenously administrated of mangiferin, which was suspended in 2 ml of dimethyl sulphoxide (10, 25, and 50 mg/kg).

Blood samples (200 μl) collected from the cervical artery at 0.5, 1.0, 1.5, 2, 2.5, 3.5, 4.5, 5.5, 7.5, 9.5, 11.5, and 24 h post-dose were seated for approximately 30 min and centrifuged at 4,000xg for 20 min to separate the plasma supernatant. To 100 μl of plasma, 100 μl of I.S. solution (2 μg/ml) and 400 μl of acetonitrile-glacial acetic acida (9:1,v:v) were added. Each tube was mixed thoroughly by vortexing for 90 s. After centrifugation for 10 min at 3,000 xg, the supernatant was transferred into labeled, clean test tubes and evaporated to dryness in a water bath at 50 °C under a stream of nitrogen. The residues were reconstituted in 100 μl of mobile phase with vortexing for 90 s, and the centrifugation procedure was repeated. Standard and QC samples were prepared following the method described above. A 20 μl aliquot was then injected onto the chromatographic column.

As to eye samples, samples from both eyes were collected at 0.5, 1.0, 1.5, 2, 2.5, 3.5, 4.5, 5.5, and 7.5 h post-dose and placed in a normal saline solution to remove blood and the conjunctiva. The eyes were then cut from the equator, and the lens and vitreous were removed. The retina was carefully dissected from the choroid and optic nerve, and the homogenate was prepared. Briefly, retina samples were weighed and homogenized in acetonitrile at 0 °C using an FJ-2000 homogenizer (Biaoben Model Factory, Shanghai, China). The homogenates were then ultrasonically extracted using a Branson Sonifier 150D (Branson, Danbury, CT) and centrifuged at 1,000× g for 10 min. The supernatants were taken out and transferred into labeled, clean test tubes. To 100 μl of supernatant, 100 μl of I.S. solution (2 μg/ml) and 400 μl of acetonitrile-glacial acetic acida (9:1, v:v) were added and mixed thoroughly by vortexing for 90 s. After centrifugation, the supernatant was transferred into labeled, clean test tubes evaporated to dryness in a water bath at 50 °C under a stream of nitrogen. The residues were reconstituted in 100 μl of mobile phase with vortexing for 90 s, and the centrifugation procedure was repeated. Standard and QC samples were prepared following the method described above. A 20 μl aliquot was then injected onto the chromatographic column.

### Assay conditions

HPLC separation was performed using the Agilent 1200 Series Rapid Resolution system. A COSMOSIL 5C_18_—MS—IIanalytical column (4.6 mm×250 mm, 5 μm, Nacalai Tesque, Inc., Kyoto Japan) was used and operated at 25 °C. The mobile phase consisted of methanol −2% glacial acetic acid (40:60,v:v) and was delivered by a G1322A pump at a flow-rate of 1.0 ml/min. The G1315BDIO diode array detector was set at 318 nm and all the measurements were performed at 35 °C. A HP1090 chromatographic work station was used for data acquisition. The sensitivity was 0.2 AUFS.

### Method validation

Method validation and documentation were performed according to guidelines set by the United States Food and Drug Administration (FDA) for bioanalytical method validation [2].

### Linearity and sensitivity

For the evaluation of the linearity of the standard calibration curve, the analyses of mangiferin in plasma and eye samples were performed on three independent days using fresh preparations. The calibration curves were prepared over a linear range of 0.50~150.00 μg/ml for plasma and 0.10~10.00 μg/ml for eye samples. Each calibration curve consisted of a double blank sample (without internal standard), a blank sample (with internal standard) and eight calibrator concentrations. Each calibration curve was constructed by plotting the analyte to internal standard peak area ratio (y) against analyte concentrations (x). The calibration curves were fitted using a least square linear regression model y=ax + b, weighted by 1/x^2^ (the reciprocal of the square of the mangiferin concentration) using the Analyst® software. The resulting a, b parameters were used to determine back-calculated concentrations, which were then statistically evaluated. All calibration curves of mangiferin were constructed before the experiments with correlation values of at least 0.995.

### Specificity

The specificity was defined as: non-interference when mangiferin was being retained from the endogenous plasma components and eye samples, and no cross-interference between mangiferin and internal standard using the proposed extraction procedure and HPLC conditions. Six different lots of blank (mangiferin-free plasma) were evaluated both with and without internal standard to assess the specificity of the method.

### Accuracy and precision

The intra- and inter-assay accuracies were expressed as the percentage difference between the measured concentration and the nominal concentration. Intra-assay precision and accuracy were calculated using replicate (n=6) determinations for each concentration of the spiked plasma sample during a single analytical run. Inter-assay precision and accuracy were calculated using replicate (n=6) determinations of each concentration made on three separate days. Accuracy (% Bias)=[(C_obs_ -C_nom_)/C_nom_] × 100. The precision (relative standard deviation; RSD) was calculated from the observed concentrations as follows: RSD=[standard deviation (SD)/C_obs_] ×100. Accuracy (Bias) and precision (RSD) values within ±15%, covering the range of actual experimental concentrations, were considered acceptable [2].

### Recovery (extraction efficiency)

The extraction efficiency of mangiferin was determined by analyzing replicate sets (n=6) of QC samples: 1.00, 10.00, and 100.00 μg/ml for rat plasma and 0.50, 2.00, and 8.00 μg/ml for rat eyes, representing low, medium and high QCs, respectively. Recovery was calculated by comparing the peak areas of mangiferin added into blank samples and extracted using the protein precipitation procedure, with those obtained from mangiferin spiked directly into post-protein precipitation solvent at three QC concentration levels.

### Stability study

The stability of mangiferin in rat plasma and rat eyes was assessed by analyzing replicates (n=6) of QC samples at concentrations of 1.00, 10.00, and 100.00 μg/ml for rat plasma and 0.50, 2.00, and 8.00 μg/ml for rat eyes. The investigation covered expected conditions during all of the sample storage and process periods, which included the stability data from freeze/thaw, bench-top, autosampler and long-term stability tests. For all stability studies, freshly prepared and stability testing QC samples were evaluated by using freshly prepared standard curves for the measurement. The concentrations obtained from all stability studies were compared with the freshly prepared QC samples, and the percentage concentration deviation was calculated. The analytes were considered stable in mouse plasma and eye when the concentration difference between the freshly prepared samples and the stability testing samples was less than 15%.

### Pharmacokinetic analysis

To evaluate the suitability of the assay for pharmacokinetic studies, 10, 25 or 50 mg/kg of mangiferin were intravenously administered to rats. Six animals were used in each dosage. Pharmacokinetic calculations were performed using the observed data. All data were subsequently processed using the DAS 2.0 program (Drug and Statistics 2.0). All values obtained were expressed in mean±standard deviation.

## Results

### Method validation

#### Linearity and sensitivity

The method was validated using the criteria described above. The data were found to be linear over a concentration range of 0.50~150.00 μg/ml in blood samples and a range of 0.10~10.00 μg/ml in eye samples. The regression equation for plasma was y=0.1637x - 0.2481, with the correlation coefficient r=0.9993, while the eye standard curve was described by the equation y=0.2832 x + 0.0280, with r=0.9994. If r>0.999, it indicated good linearity. The limit of quantitation (LOQ) was 0.50 μg/ml for plasma and 0.10 μg/ml for eye, which can be determined with a relative error (RE) and precision (RSD) of <20% at a signal-to-noise ratio of 3. The limits of detection (LOD) were 0.20 μg/ml and 0.10 μg/ml for plasma and eye, based on a signal to noise ratio of 3.

#### Specificity

**Figure 2:**
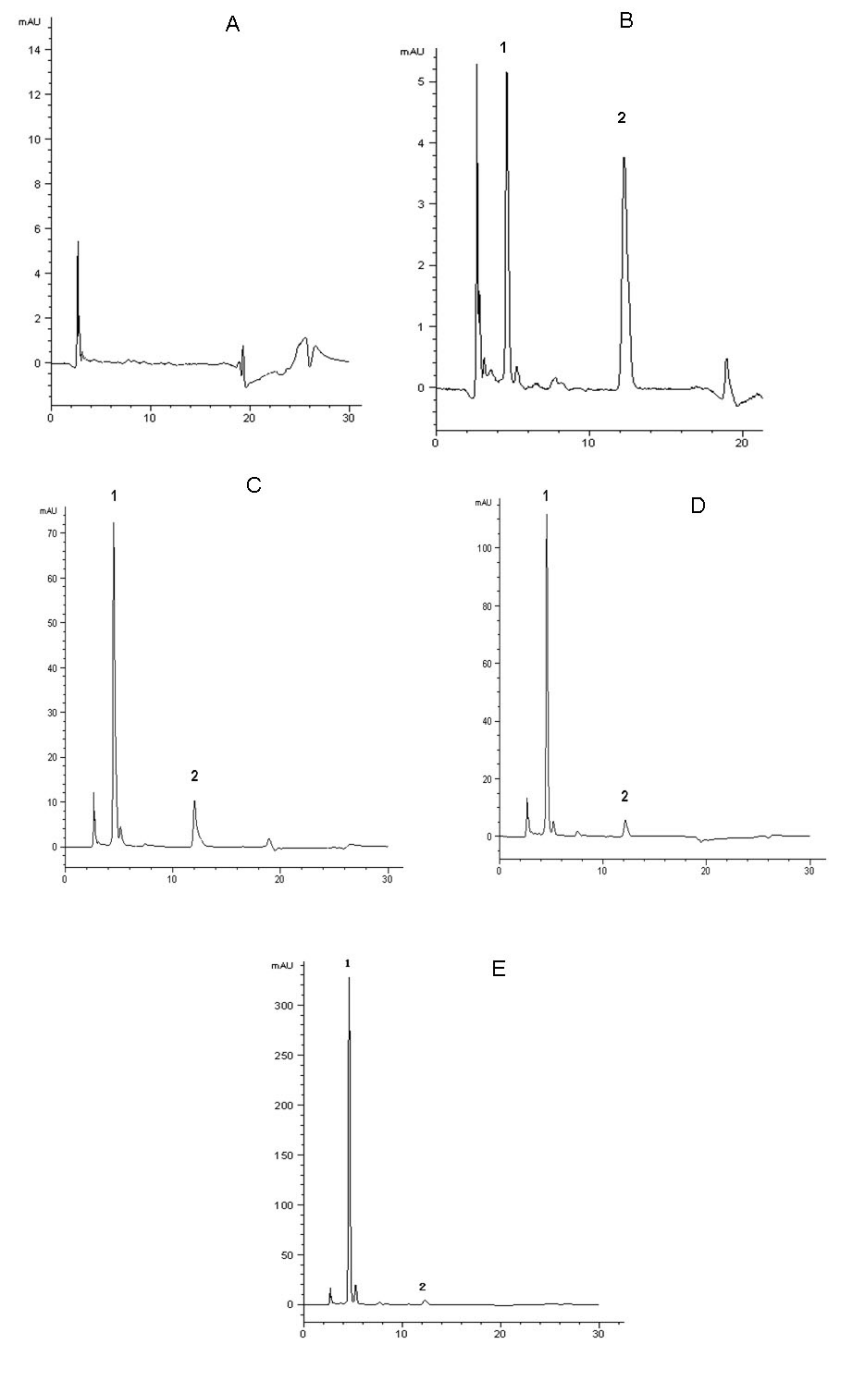
HPLC chromatograms of plasma mangiferin. HPLC separation was performed on plasma samples using the Agilent 1200 Series Rapid Resolution system. A COSMOSIL 5C_18_—MS—IIanalytical column (4.6 mm×250 mm,5 μm) was used and operated at 25 °C. The mobile phase consisted of methanol −2% glacial acetic acid (40:60 v:v). Typical chromatograms of blank plasma, blank plasma spiked with mangiferin and I.S., and rat plasma sample after injection of mangiferin are presented. Mangiferin and the I.S. were eluted at 5.6 and 12.16 min, respectively. The total run time was less than 30 min. A good separation of the I.S. and mangiferin was obtained under the specified chromatographic conditions. There is no disturbance from the background signals in the plasma after the protein precipitation step. **A**: Typical chromatogram of blank plasma. **B**. Typical chromatogram of blank plasma spiked with standard mangiferin (5 μg/ml) and I.S. panel. **C**. Typical chromatogram of blood sample containing mangiferin (24.14 µg/ml) collected at 0.5 h after mangiferin administration (10 mg/kg, i.v.). **D**: Typical chromatogram of blood sample containing mangiferin (73.88 µg/ml) collected at 0.5 h after mangiferin administration (25 mg/kg, i.v.). **E**: Typical chromatogram of blood sample containing mangiferin (221.54 µg/ml) collected at 0.5 h after mangiferin administration (50 mg/kg, i.v.).

**Figure 3:**
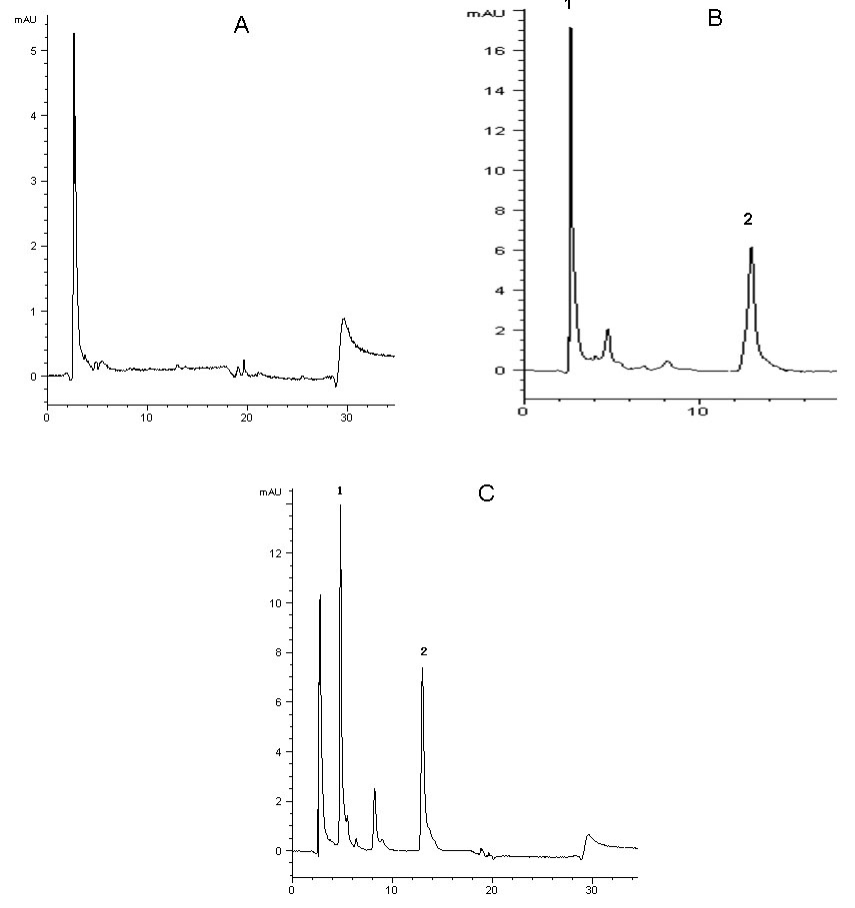
HPLC chromatograms of mangiferin in the eye. HPLC separation was performed using the Agilent 1200 Series Rapid Resolution system. A COSMOSIL 5C_18_—MS—IIanalytical column (4.6 mm×250 mm, 5 μm) was used and operated at 25 °C. The mobile phase consisted of methanol −2% glacial acetic acid (40:60,v:v). Typical chromatograms of blank eye, blank eye spiked with mangiferin and I.S., and rat eye sample after injection of mangiferin are presented. Mangiferin and the I.S. were eluted at 5.6 and 12.16 min, respectively. The total run time was less than 30 min. A good separation of the I.S. and mangiferin was obtained under the specified chromatographic conditions. There is no disturbance from the background signals in the eye after the protein precipitation step. **A**: Typical chromatogram of blank eye sample. **B**: Typical chromatogram of blank eye sample spiked with standard mangiferin (1 μg/ml) and I.S. **C**: Typical chromatogram of eye sample containing mangiferin (5.63 µg/ml) collected at 1 h after mangiferin administration (50 mg/kg, i.v.).

Under this chromatographic condition, the number of theoretical plates was 7,000 and the resolution of mangiferin was 115 peaks/min. The degree of interference by endogenous plasma or eye constituents with mangiferin and I.S. was assessed by inspection of chromatograms derived from a processed blank plasma sample or blank eye sample. Typical chromatograms of blank plasma, blank plasma spiked with mangiferin and I.S. and rat plasma sample after injection of mangiferin are presented in Figure 2. Typical chromatograms of blank eye sample, blank eye sample spiked with mangiferin and I.S. and rat eye sample after injection of mangiferin are presented in Figure 3. Mangiferin and I.S. were eluted at 5.6 and 12.16 min, respectively. The total run time was less than 30 min. A good separation of I.S. and mangiferin was obtained under the specified chromatographic conditions. There is no disturbance from the background signals either in the plasma or the eye samples after the protein precipitation step, which can be seen from Figure 2 and Figure 3.

#### Accuracy, precision, recovery, and stability

The accuracies for intra- and inter-day assay, the recoveries and the stability of mangiferin were estimated at three concentrations (1.00, 10.00, and 100.00 μg/ml for rat plasma and 0.50, 2.00, and 8.00 μg/ml for rat eye). As shown in Table 1, the intra-day and inter-day precisions (% RSD) in rat plasma and eye were <15%. The recovery was approximately 82.47%–93.34% in the plasma and 90.91%–97.00% in the eye, as is shown in Table 2. The stability of mangiferin are shown in Table 3. The precision for freeze/thaw samples ranged from 3.81−8.26% for plasma and 2.72%–9.38% for eye, and the accuracy ranged from 102.01−110.25% for plasma and 99.56%–108.75% for eye, respectively. The results indicated that mangiferin was stable in either plasma or eye for three cycles when stored at −20 °C and thawed to room temperature. The precision for bench-top stability ranged from 3.32−8.54% for plasma and 7.48%–10.26% for eye, and the accuracy ranged from 90.69−99.58% for plasma and 94.29%–98.35% for eye, respectively. This indicates reliable stability under the experimental conditions of the analytical runs. The precision and accuracy for long-term stability samples ranged from 3.84−7.92% and 92.51%–101.38% for plasma and 4.03−8.25% and 99.16%–105.54% for eye, respectively. The results of long-term storage stability data indicated that the plasma and eye samples were stable at −20 °C for over 1 month. The precision for the autosampler stability ranged from 4.97%–6.31% for plasma and 2.38%–6.43% for eye, and the accuracy ranged from 89.35%–92.59% for plasma and 91.24%–96.67% for eye, respectively. The result suggested that mangiferin could be analyzed for over 8 h in the autosampler tray at 4 °C with acceptable levels of precision and accuracy. The results of stability experiments showed that no stability-related problems occurred during sample storage, extraction or chromatography processes for mangiferin in plasma and eye samples.

**Table 1:** Intra- and inter-day precision and accuracy of mangiferin in rat plasma and eye (n=6).

**Matrix**	**Nominal concentration (μg/ml)**	**Observed concentration (μg/ml)±SD**	**Accuracy (% bias)**	**RSD (%)**
Plasma Intra-day (n=6)	1	0.96+0.08	−4	8.33
	10	9.89±0.51	−1.1	5.15
	100	99.62±6.80	−0.4	6.82
Inter-day(n=6)	1	0.94±0.08	−6	8.51
	10	10.02±1.01	2	10.31
	100	99.48±0.08	−0.5	0.08
Eye Intra-day(n=6)	0.5	0.49±0.04	−2	8.16
	2	2.02±0.18	2	9.09
	8	7.97±0.19	−0.4	2.38
Inter-day(n=6)	0.5	0.48±0.04	−4	8.33
	2	1.95±0.23	−2.5	11.79
	8	8.01±0.08	0.1	1.01

The intra- and inter-assay accuracies were expressed as the percent difference between the measured concentration and the nominal concentration. Intra-assay precision and accuracy were calculated using replicate (n=6) determinations for each concentration of the spiked plasma sample during a single analytical run. Inter-assay precision and accuracy were calculated using replicate (n=6) determinations of each concentration made on three separate days. Accuracy (% Bias)=[(C_obs_ -C_nom_) /C_nom_]×100.The precision (relative standard deviation; RSD) was calculated from the observed concentrations as follows: RSD=[standard deviation (SD) / C_obs_] ×100. Accuracy (Bias) and precision (RSD) values within ±15% covering the range of actual experimental concentrations were considered acceptable [2].

**Table 2:** Recovery of mangiferin from rat plasma and eye (n=6).

**Matrix**	**Spiked concentration (μg/ml)**	**Recovery (%±SD)**	**RSD (%±SD)**
Plasma	1	0.825±0.05	6.4
	10	0.832±0.11	13.6
	100	0.931±0.15	14.9
Eye	0.5	0.911±0.24	5.8
	2	0.969±0.06	7
	8	0.923±0.14	14.7

The extraction efficiency of mangiferin was determined by analyzing replicate sets (n=6) of QC samples: 1.00,10.00, and 100.00 μg/ml for rat plasma and 0.50, 2.00, and 8.00 μg/ml for rat eye, representing low, medium, and high QCs, respectively. Recovery was calculated by comparing the peak areas of mangiferin added into blank samples and extracted using the protein precipitation procedure with those obtained from mangiferin spiked directly into post-protein precipitation solvent at three QC concentration levels.

**Table 3:** Stability data for mangiferin under various conditions (n=6)

**Tissue**	**Storage period and storage condition**	**Nominal Concentration (µg/ml)**	**Observed concentration (µg/ml)±SD**	**Accuracy (%)**	**RSD (%)**
Plasma	3 Freeze/thaw cycles(−20 °C)	1	1.06±0.06	107.56	8.26
		10	10.9±0.24	110.25	3.81
		100	102.12±5.30	102.01	5.65
	Process sample stability (RT; 8 h)	1	0.98±0.65	99.58	8.54
		10	9.86±0.73	98.75	3.32
		100	90.48±0.59	90.69	7.15
	Long-term stability (−20 °C; One month)	1	0.96±0.08	97.18	7.92
		10	9.19±0.84	92.51	6.39
		100	100.82±0.13	101.38	3.84
	Autosampler stability (4 °C; 8 h)	1	0.91±0.31	91.44	5.24
		10	9.31±0.74	92.59	6.31
		100	89.43±0.21	89.35	4.97
Eye	3 Freeze /thaw cycles (−20 °C)	0.5	0.50±0.94	108.75	9.38
		2	1.98±0.54	99.56	3.45
		8	8.01±0.16	102.79	2.72
	Process sample stability (RT; 8 h)	0.5	0.46±0.06	95.37	8.68
		2	1.97±0.32	94.29	10.26
		8	7.98±0.16	98.35	7.4
	Long-term stability (−20 °C; One month)	0.5	0.51±0.29	105.54	6.51
		2	2.02±0.08	101.32	8.25
		8	7.99±0.11	99.16	4.03
	Autosampler stability (4 °C; 8 h)	0.5	0.47±0.69	91.24	2.38
		2	1.96±0.37	96.67	4.76
		8	7.87±0.46	92.54	6.43

The stability of mangiferin in rat plasma and eye were assessed by analyzing replicates (n=6) of QC samples at concentrations of 1.00, 10.00, and 100.00 μg/ml for rat plasma and 0.50, 2.00, and 8.00 μg/ml for rat eye. The investigation covered expected conditions during all of the sample storage and process periods, which included the stability data from freeze/thaw, bench-top, autosampler and long-term stability tests. For all stability studies, freshly prepared and stability testing QC samples were evaluated by using freshly prepared standard curve for the measurement. The concentrations obtained from all stability studies were compared with the freshly prepared QC samples, and the percentage concentration deviation was calculated. The analytes were considered stable in mouse plasma and eye when the concentration difference was less than 15% between the freshly prepared samples and the stability testing samples.

### Application to pharmacokinetic study

**Figure 4:**
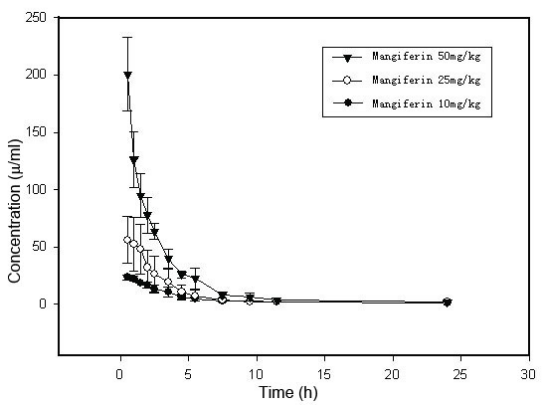
Mean level of mangiferin in rat blood after mangiferin administration (10, 25, and 50 mg/kg, i.v.). Data are expressed as mean±SEM (n=6). The concentration-time data after a single intravenous administration of 10, 25, or 50 mg/kg showed that the major parameters were correlated positively with the dosage given. The mean plasma concentration–time profile is illustrated here.

The sampling technique and liquid chromatographic detection were then applied to the pharmacokinetic characterization using the DAS 2.0 program (Drug and Statistics 2.0, Hefei, China). After a single intravenous administration of 10, 25, or 50 mg/kg of mangiferin, the concentration-time data conformed to a two-compartment model and the major parameters correlated positively with the dosage given. The dosages for Cmax were 24.75±0.85, 56.77±21.13, and 200.77±32.12 μg/ml, respectively, for AUC(0-t), they were 121.48±12.92, 205.28±104.30, and 577.71±63.30l μg/ml/h, respectively. The pharmacokinetic parameters are listed in Table 4. The mean plasma concentration–time profile is illustrated in Figure 4. Results of the study are consistent with the works by Lai [17] and with other previously published studies [12–16]. Detectable mangiferin was found in the eye, as shown in Table 5.The mangiferin concentration reached 5.69±1.48 μg/ml 0.5 h after intravenous administration (50 mg/kg) and then dropped gradually to 0.30±0.02 μg/ml 5.0h later. The pharmacokinetic parameters of the retina are listed in Table 6.

**Table 4:** Pharmacokinetic parameters of plasma following mangiferin administration (10, 25, and 50 mg/kg, i.v.) by Drug and statistics 2.0

**Parameters**	**10 mg/kg**	**25 mg/kg**	**50 mg/kg**
T_max_ (h)	0.75±0.27	0.58 ±0.20	0.50±0.01
C_max_ (μg/ml)	24.75±0.85	56.77±21.13	200.77±32.12
t_1/2α_ (h)	2.15±0.81	1.60±0.28	0.81±0.60
t_1/2β_ (h)	3.12±0.04	1.61±0.31	3.25±1.82
AUC_0-t_ (μg/ml·h^−1^)	121.48±12.92	205.28±104.30	577.71±63.30
K_21_ (h^−1^)	0.09±0.10	0.37±0.19	0.60±0.57
K_10_ (h^−1^)	0.18±0.01	0.35±0.05	0.58±0.37
K_12_ (h^−1^)	0.17±0.10	0.22±0.25	1.28±1.90
MRT_0-t_ (h)	5.89±0.06	4.23±0.66	3.10±0.23
CL (L/kg·h^−1^)	0.06±0.01	0.13±0.06	0.07 ±0.01

The concentration-time data after single intravenous administration of 10, 25 or 50 mg/kg of mangiferin conformed to a two-compartment model. **Tmax:** time of the maximum plasma concentration; **C_max_**: maximum plasma concentration following drug administration; **t_1/2(α)_**: distribution half-life; **t_1/2(β)_**: elimination half-life; **AUC**: area under the plasma concentration–time curve; **K_10_:** first order elimination from compartment 1; **K_12_:** first order transfer from compartment1 to compartment 2; **K_21_:** first order transfer from compartment 2 to compartment 1; **MRT**, mean residence time. **Cl**, clearance;

**Table 5:** Mean level of mangiferin in rat retina after mangiferin administration (50 mg/ kg, i.v.). Data are expressed as mean±SEM (n=6).

**Time (h)**	**Concentration (μg/ml)**
0.5	5.69±1.48
1	5.63±1.30
1.5	3.70±1.13
2	3.63±0.06
2.5	2.96±0.31
3.5	1.83±0.06
4.5	1.36±0.02
5.5	0.30±0.02

Detectable mangiferin was found in the eye as shown in Table 5.The mangiferin concentration reached 5.69±1.48 μg/ml 0.5 h after intravenous administration (50 mg/kg) and then dropped gradually to 0.30±0.02 μg/ml 5.0 h later.

**Table 6:** Pharmacokinetic parameters of retina following mangiferin administration (50 mg/kg, i.v.) by Drug and statistics 2.0

**Paremeters**	**Unit**	**Average**
t_1/2α_	h	1.87±0.40
t_1/2β_	h	1.87±0.40
V1	L/kg	8.14±1.28
CL	L/h/kg	2.52±0.38
AUC(0-t)	Mg/L·h-1	15.64±1.98
MRT(0-t)	h	1.86±0.07
K_12_	1/h	0.18±0.19
K_21_	1/h	0.39±0.09

The concentration-time data after single intravenous administration of 50 mg/kg of mangiferin conformed to a two-compartment model. **t_1/2(α)_**: distribution half-life; **t_1/2(β)_**: elimination half-life; **V1**:Volume of distribution; **Cl**, clearance;**AUC**: area under the plasma concentration–time curve; **MRT**, mean residence time; **K_12_:** first order transfer from compartment1 to compartment 2; **K_21_:** first order transfer from compartment 2 to compartment 1.

## Discussion

For the first time, our study showed that mangiferin can cross the blood-retina barrier after a single intravenous administration. The study also showed that the HPLC method is suitable for detecting mangiferin in the eye.

HPLC has been widely used in the detection of mangiferin in herbs as well as biologic fluids [12,13,15,16,18,19]. However, whether it is applicable to the pharmacokinetic study of mangiferin in the eye remains unknown. The limit of quantification (LOQ) was defined as the lowest drug concentration that can be determined with a relative error (RE) and precision (RSD) of <20% at a signal-to-noise ratio of 3. In practice, this is the lowest concentration in the calibration curve. The LOQ in this study was 0.50 μg/ml for plasma and 0.10 μg/ml for eye, respectively. The LOQ for both eye and plasma samples was sufficient for pharmacokinetic studies of mangiferin. The eye tissue contains an abundance of protein, and thus the deproteinization is important in the pre-treatment of eye tissues. In this study, we tried pre-treating the eye sample using the standard method, but results showed that an extremely low amount of mangiferin was detected.

A further study indicated that most of the compound remained in the eye tissue, indicating that mangiferin may possess a high protein-binding affinity. Therefore, we adopted homogenization and ultrasonic extraction with acetonitrile to obtain a high amount of mangiferin from the eye tissue. Acetonitrile-acetic acid (9:1, v/v) was then used in protein-precipitating to pre-treat both the plasma and eye samples before HPLC. The background signal of plasma or eye had little or no effect on the HPLC separation conditions after the simple protein precipitation clean-up step. The above results have shown that HPLC is an appropriate method to study the pharmokinetic parameters of mangiferin in the eye with adequate sensitivity, selectivity and accuracy.

The blood-brain barrier (BBB) is a separation of circulating blood and cerebrospinal fluid (CSF) maintained by the choroid plexus in the central nervous system (CNS) [20]. Much like the brain, the eye has evolved cellular barriers to exclude potentially harmful blood-borne agents while allowing passage of nutrients to ensure proper functioning. The barrier is known as the blood-retina barrier (BRB), which separates the retina and other contents from the fenestrated vascular system of the neighboring choroid [21]. Lai et al. have shown that when 10, 30, and100 mg/kg of mangiferin was administered intravenously, the parent molecule of mangiferin was not detected in the brain regions for any of the dosage treatments. This finding suggested that mangiferin cannot cross the BBB or is restricted by its detection limit [17]. Another study by Li et al. [22] proved that mangiferin could pass through the BBB and showed pharmacological activity, despite a low level after a single oral dose of 15 g/kg *Rhizoma anemarrhenae* extract. Martinez et al. also demonstrated that mangiferin was able to traverse the BBB [23]. It has also been shown that mangiferin has real potential to ameliorate the oxidative stress observed in neurodegenerative disorders. Mangiferin (20 mg/kg, p.o.) has been proven to improve long-term cholinergic memory deficits by AChE inhibition or cholinergic receptor stimulation and inhibition of NF-κB activation in vivo [24]. All these studies supported the fact that mangiferin can penetrate the BBB and play a protective role in neurologic diseases. However, whether mangiferin can pass the BRB remains unknown.

In contrast to the BRB, the BBB is established by endothelial cells rather than by epithelial cells. The BRB consists of the inner blood–retina barrier (iBRB), which is formed by elaborate capillaries with barrier characteristics, and the outer blood–retina barrier (oBRB), which is formed by the retinal pigment epithelium (RPE). The oBRB separates the neural retina from the fenestrated vascular system of the neighboring choroid [21]. Studies based on a broad range of test agents have provided evidence that the epithelial BRB mirrors the pharmacological properties of the endothelial BBB to a large extent, and expresses the same major efflux systems. The permeation ranking of the selected compounds was similar in the BRB and BBB in vitro system. Our results showed that mangiferin can be found in the eye, and that the concentration in the vitrous went down with the plasma. Mangiferin in the retina reached 5.69±1.48 μg/ml 0.5 h after intravenous administration (50 mg/ kg mangiferin), and the eye-to-plasma concentration ratio of mangiferin was 2.80%. Additionally, we have also proven that 5 μg/ml mangiferin can exert an anti-oxidant effect in the retina (Unpublished data).

### Conclusion

Because of the blood-retina barrier, a study of whether a drug can reach the target tissue in the eye will play an important role in illustrating the barrier’s mechanism and investigating the impact of the drug. The results of the present study are useful for the administration of mangiferin in eye diseases and understanding mangiferin’s pharmacokinetic behavior.
